# Analysis of foot and ankle disorders and prediction of gait in multiple sclerosis rehabilitation

**DOI:** 10.1186/s40001-014-0073-5

**Published:** 2014-12-24

**Authors:** Ligia Rusu, Marius Cristian Neamtu, Eugenia Rosulescu, Germina Cosma, Mihai Dragomir, Mihnea Ion Marin

**Affiliations:** Sports Medicine and Kinesiology Department, University of Craiova, Srada Alexandru Ioan Cuza 13, Craiova, 200585 Romania; Department of Pathologic Physiology, University of Medicine and Pharmacy of Craiova, Strada Petru Rareş 2-4, Craiova, 200349 Romania; Department of Theory and Methodology of Motricity Activities, University of Craiova, Strada Alexandru Ioan Cuza 13, Craiova, 200585 Romania; Department of Applied Mechanics, University of Craiova, Strada Alexandru Ioan Cuza 13, Craiova, 200585 Romania

**Keywords:** Assessment, Biomechanical, Foot, Gait, Motor control

## Abstract

**Background:**

Multiple sclerosis (MS) is a disease of the central nervous system probably based on the autoimmune mechanism against myelin and the action of lymphocyte T. In the last 50 years, more than 150 descriptive studies regarding MS have focused on the etiopathogeny, treatment, diagnosis and prevention of the progressive evolution of MS. Most recently, studies in the field of rehabilitation and diagnosis have tried to present the postural aspects of control/foot and ankle control and gait pattern in MS. The aim of this study is focused on biomechanical foot analyses of MS patients.

**Methods:**

Our clinical research and functional assessment was based on a scale like the EDSS/Kurtzke score: biomechanical foot assessment used the RSscan force plate to assess the foot loading, impulse and foot-ankle angle (subtalar angle), and pressure distribution methods for statistical analyses. The study included MS patients at the Neurologic Rehabilitation Unit, Craiova, we studied 48 patients (46.04 ± 10.99 years) diagnosed with MS.

**Results:**

This study shows that the major lesion is to the pyramidal system and the average value for functionality index (EDSS score) is 3.03 ± 0.13, where 3 means easy paraparesis or hemiparesis. In considering postural strategies, we observed an instability left to right to be more evident in the swing phase and it influences the under the foot impulse for the next step and postural control. From the analysis of the data and pressure centre position, we can see that the high pressure is on metatarsian II to III and more or less at the heel. This means the development of an ankle strategy necessary to restore balance, stability and motor control cannot be assessed other than by clinical evaluation.

Even if many physicians and physical therapists do use the functional scale in their daily assessment, it does not help us achieve a complex assessment of gait and lower limb behaviour during gait, nor does it provide information about the impact of gait on daily activities and on quality of life.

**Conclusions:**

Biomechanical assessment can help the clinician predict the functional evolution of MS patients without visible clinical gait disorders and allows the development of a strategy for rehabilitation to prevent an incorrect ankle/ankle and foot position, resulting in a lack of motor control.

## Background

Multiple sclerosis (MS) is a diseases of the central nervous system probably based on the autoimmune mechanism against myelin and the action of lymphocyte T. MS affects more people in Europe [1], and in the last 50 years, more than 150 descriptive studies have been developed regarding MS, focused on the etiopathogeny, treatment, diagnosis and prevention of the progressive evolution of MS. Most recently, studies in the field of rehabilitation and diagnosis have tried to present aspects of postural control, foot control and gait pattern in MS. Some reports describe the gait pattern in MS using biomechanical assessment and analyse the temporal distance parameters and assess the ankle joint range of motion. One of these studies focused on performing a biomechanical characterisation of gait patterns among people with MS. This study describes typical gait patterns of people with MS and common pathways in the degeneration of ambulatory ability as a consequence of disease progression. All of this information helps in the design and purpose of a rehabilitation programme [2].

Other problems for MS patients are postural, foot control during gait and orthostatism, because of muscle fatigue [3,4], spasticity [5] or decrease of muscle strength [6]. In all of these studies, the authors proposed an evaluation of the pressure deviation centre [7-9].

Based on these aspects of research and the questions arising, we propose to make an algorithm for a complex assessment in MS, which can be useful for the prevention of motor disorder evolution and for building the goals of rehabilitation programmes. From this point of view, the aim of this research is to evaluate the biomechanical foot parameters of MS patients during gait.

Hypothesis: this study is based on research on the assessment of MS patients and presents the results of biomechanical assessment of foot during gait analyses with the aim of improving rehabilitation programmes.

The aim of this study is focused on biomechanical foot analyses of MS patients, because this assessment can improve the evaluation of patients and can help predict their long-term evolution.

Clinical assessment is the first step and it is made in accordance with the McDonald criteria, based on the dissemination of inflammatory injuries of the nervous system in time and space.

The study is based on the following problems: how balance and the foot operate; does postural control influence gait; what is the clinical and functional status of MS patients who participate in physical therapy programmes; and what are the specific biomechanical parameters of the foot in MS patients.

## Methods

### Study design

The study includes MS patients, with reference to the McDonald criteria for patients with primary progressive multiple sclerosis (PPMS), with ≥1 year of disease progression, brain dissemination in space, and clinical certainty of MS.

The study participants are patients in the Neurologic Rehabilitation Unit, Craiova. The assessment is based on mutidisciplinary evaluation regarding clinical and functional assessment. We studied 48 patients (46.04 ± 10.99 years) diagnosed with mild clinical forms of MS (clinical certainty of MS). From 48 subjects: 25 have no gait disorders (52.08%), 16 subjects (33.33%) have minor/mild gait disorders expressed by dynamic balance disturbance (they need assistance or need devices, but they can follow gait cycles and perform daily activities), 7 subjects (14.58 %) have no ambulation. From all of these, only seven subjects did not participate to the study, because of no ambulation.

### Sample inclusion criteria

Sample inclusion criteria were: MS form according to the McDonald criteria; aged more than 18 years; motor disorders from 0 to 8 according to the Expanded Disability Status Scale (EDSS); and sensitive disorders, ataxia, chronic fatigue, or vertigo.

### Sample exclusion criteria

Sample exclusion criteria were: pregnancy; patient refusal; one or more spikes of the clinical aspects in the last three months; cognitive disorders; associated pathology like cardiorespiratory disease, orthopaedic disease or other neuromotor pathology besides MS or visual disorders (optic nevritis).

### Evaluation

#### Clinical evaluation

Clinical evaluation included a complete neurological examination (muscle strength, presence of pyramidal signs, evaluation of gait disturbance, sensorial disorders and the presence of cerebellar signs, and assessment of visual and auditory acuity) [10-13]. To evaluate cognitive problems, we used the mini-mental state examination (MMSE). In this way, we excluded patients that scored less than 27 points.

#### Functional evaluation

Functional evaluation was achieved by conformity with the EDSS/Kurtzke, and the Impairment, Disabilities, and Handicap (IDH) scale score of movement capacity [14-16].

#### Biomechanical foot evaluation

Biomedical foot evaluation was performed using a FootScan Scientific Version planting force plate (RSscan International, Olen, Belgium) for assessing the force distribution and plantar pressure distribution, which was able to perform measurements with a frequency of 500 Hz in two dimensions and record the complete action of both plants. The platform was used to record the pressure distribution values in the lower limb at ground contact. The plant applied on the platform measured local pressure at full contact with the ground at high frequency; the operational substrate is represented by the total impact force measured at the level of a sensor matrix on a known surface [17].

**Figure 1:**
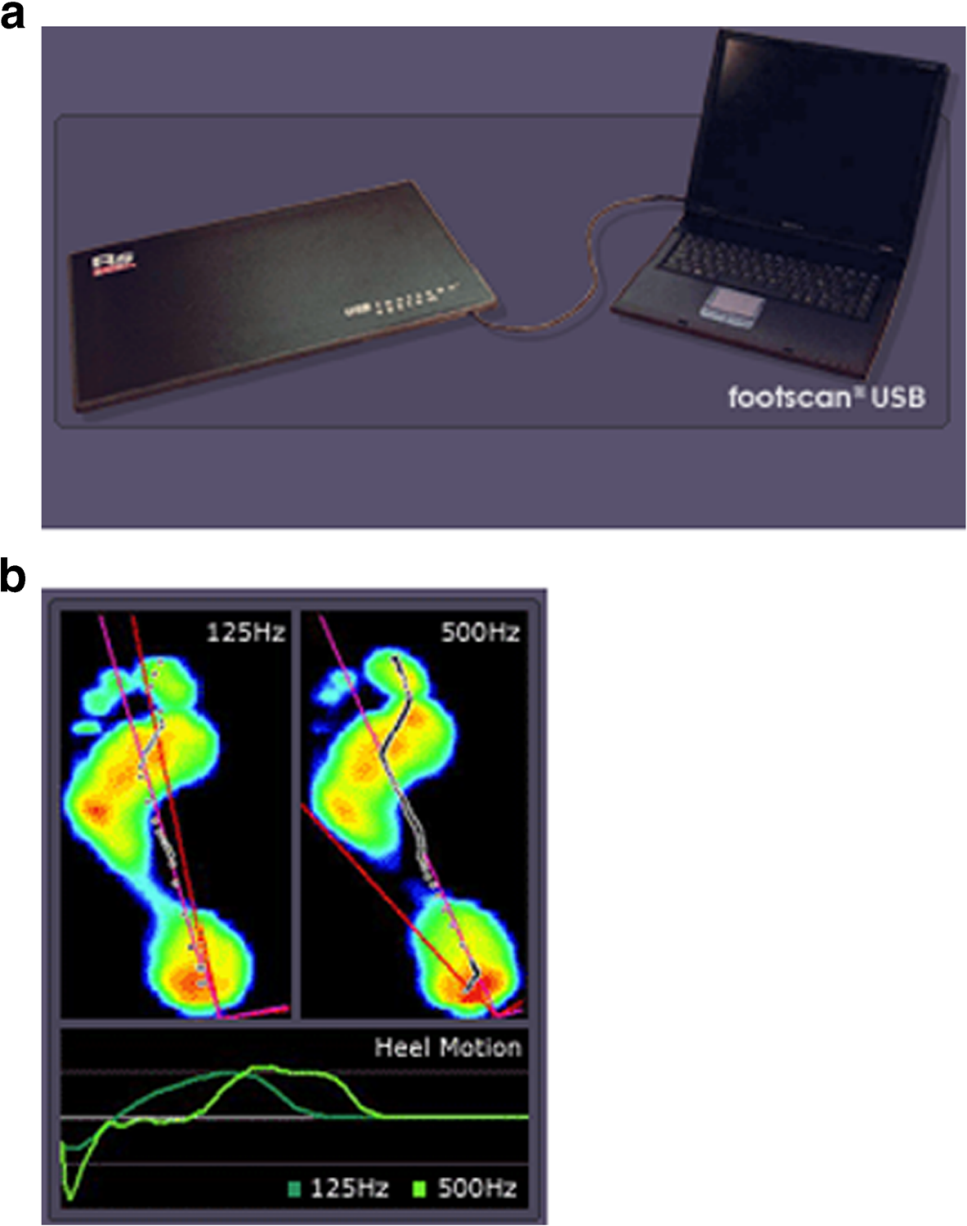
**Components of force plate RSscan -plate, connection with PC (a) and plantar profile during gait recording by force plate (b).**

An RSscan force platform (Figure 1a, b) recorded the pressure and force developed during gait phases. The values are expressed in N for force and N/cm^2^ for pressure. These measurements allow the study of the lower limb during gait, with or without assistive means. Data analysis includes: information about pressure distribution at plantar level, depending on time; force distribution in each plantar region, depending on time; load values in each region; the contact surface that is active (in direct contact with the platform surface, which stimulates the platform sensors); limbs axis and subtalar angle; limb balance in anteroposterior and frontal planes, and pressure centre position.

Seven of the subjects (six in wheelchairs and one without ambulation) had not participated in this type of investigation previously.

**Figure 2:**
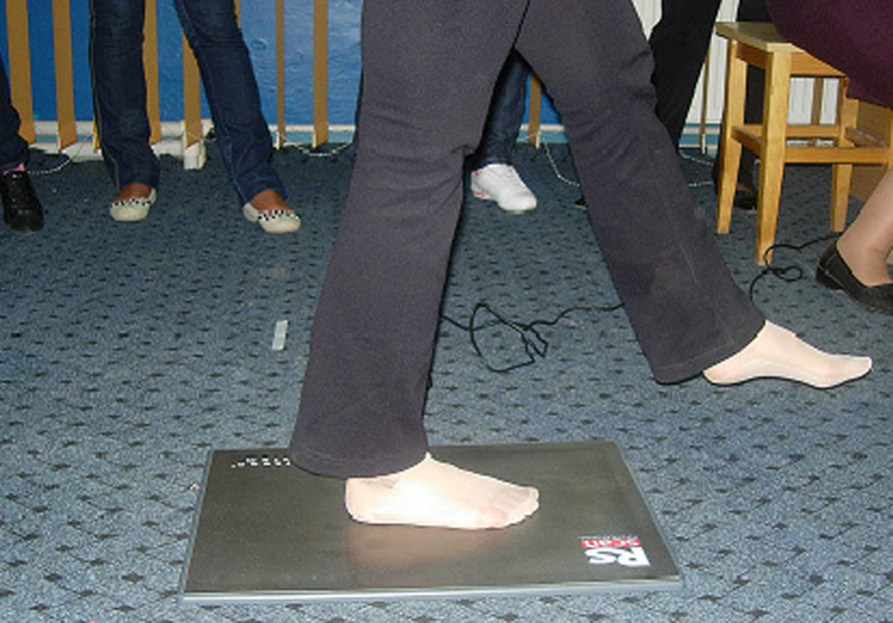
**Recording on the force plate.**

Both plants were recorded during two gait cycles, paying attention to alternative placement of the right and left lower limb (Figure 2). We made three assessments and we chose the best. The distance for gait analyses was 2.50 metres (1 m before the force plate, 50 cm on the force plate, and 1 m after the force plate); the force plate was placed 1 metre from the start line.

In the present study, we grouped the eight stages of gait into three stages, namely: heel attack phase - the initial contact heel; midstance phase, in which the middle region of the plant is involved; and the swing phase, in which the load is higher in the metatarsals, this stage depends on the way tibial-tarsal control is achieved.

The parameters studied were:

- Impulse (I) (Ns/cm) is the total load of the assessed region.
- Loading rate measured in the region, load rate (LR) (N/cms), which is the upload speed in the assessed region
- Contact area (CA) (cm^2^) is the area corresponding to each evaluated area
- Pressure distribution during gait relative to the sustaining surface
- Graphs of foot balance (that is information about heel rotation, foot balance, and load in the metatarsal area, which provides information about foot stability during gait)
- Foot angle between foot and walking direction, which can be positive (that is exorotation), or negative (that is endorotation)
- Subtalar angle between the talus and calcaneus. The measurement was taken after the patient understood how to work the system, at constant walking speed. Anamnestic information included: name, height, weight, and foot size.

Data collection: the plantar level was recorded at four sites: lateral heel (HL); medial heel (HM); midfoot (MF); and toes II to V.

We chose these sites because they are involved in the stance and propulsion gait phases; in addition, these plantar sites need much more motor control. Our recording included both feet.

#### Paraclinical evaluation

The paraclinical evaluation used magnetic resonance imaging (MRI) and found focal degenerative lesions.

### Statistical analyses

For statistical analysis we used Microsoft Excel computer software (Microsoft, Redmond, WA, USA) and academic-specific Minitab version 15 (Minitab Inc, State College, PA, USA) or EPI 2000 software (CDC, Atlanta, GA, USA).

Predefined functions in Microsoft Excel were used for the data analysis module, for example, XLSTAT and WinSTAT.

Experimental data were transferred to a Microsoft Office Excel workbook, where we created a database to extract the significant dates for this study.

Data analysis was carried out using Microsoft Excel and SPSS 16.0 (SPSS Inc, Chicago, IL, USA) software programs. The descriptive analysis included: median (percentile 25 to 75%) and media. The analytical analysis included percentage (x%) and Student’s *t* statistic between experimental group values and values stored in the RSscan’s software. *P* ≤0.05 was considered of statistical significance for clinical and functional assessment.

The research was carried out in compliance with ethical principles, and the Declaration of Helsinki Law No. 206/2004. The research is approved by the Ethics Committee of the University of Craiova, and also we obtained informed consent from all patient participants.

## Results

### Clinical, functional and paraclinical assessment

All patients had confirmed MS based on clinical aspects and MRI result meaning periventricular lesions, juxtacortical lesions and spinal lesions.

- ***Demographic features***
  Gender (Table 1)

**Table 1:** **Gender distribution of patients with MS**

**Gender**	**Number (%) patients**
Female	32 (67%)
Male	16 (33%)
TOTAL	48 (100%)
  We observed a prevalence of female patients - 32 (67%) versus 16 male (33%), (*P* <0.01), this is in accordance with the relevant literature regarding the prevalence of MS among females.
- ***Age and debut of MS***
  The time lapse from debut until the research activity was 2 to 23 years (11.13 ± 5.57 years).
- ***Motor disorders***
  There were gait disorders due to sensorial disturbance in 23 MS subjects (47.92%), 25 MS patients (52.08%) were without gait disorders (Table 2).

**Table 2:** **Gait disorders and functional independence**

***Gait***	**Multiple sclerosis patients, n = 48**	
	**Number**	**%**
Without gait disorders	25	52.08
300 m without stance device	7	14.58
200 m without stance device	1	2.08
100 m without stance device	2	4.16
100 m needing help and assistance	4	8.33
20 m permanent assistance	2	4.16
Wheelchair	6	12.5
No ambulation	1	2.08
- ***Scale EDSS/Kurtzke***

The EDSS/Kurtzke scale was used to allow the integration of the patients’ dependence on functional parameters and it is between 0 and 10. The MS patients with gait disorders and those who use assistive devices, scored 6 to 7.5 representing 33.33% and MS patients with total assistance requirements and no ambulation scored 7 to 10, representing 14.58%, 52.08% were without gait disorders.

**Figure 3:**
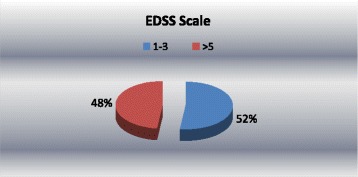
**Expanded Disability Status Scale distribution.**

The total EDSS/Kurtzke score for MS patients was 3.27 ± 0.15. The distribution of this score is presented in Figure 3.

Muscle-force testing (scale 0 to 5) shows a decrease in muscle force (F = 2), and the functional evaluation shows the presence of pyramidal signs highlighted by bilateral motor deficit in the lower limbs, sensory disturbances such as hyperaesthesia, ataxic gait, with no changes in visual and auditory acuity. The evolutionary stage is of primary chronic progressive multiple sclerosis.

### Biomechanical assessment

Biomechanical analysis was conducted on three important gait phases ordered by the nervous system, motor control and how the muscle contraction can be initiated.

The parameters recorded using the RSscan force plate were: contact area (CA) (cm^2^), for each region of plantar level, reporting on plantar pressure (P) and maximal force (Fmax) presented in the next Tables 3, 4, 5, 6. Average values for the contact area are presented in Table 7.

**Table 3:** **Lateral heel**

***Statistical values***	***Contact area*** **(** ***CA*** **)/** ***Pressure*** **(** ***P*** **)**		**Contact area (CA)/Force (F)**	
	**Left**	**Right**	**Left**	**Right**
Media	64.95	68.15	66.1	66.565
Standard deviation (SD)	18.68711	17.29093	18.75577	21.57619
Min	12	40	13	10.3
Max	94	97	95	98
No. values	20	20	20	20
***P*** **(Student’s** ***t*** **) for contact area (CA)**	0.398786	0.499235	0.456442	0.479734

**Table 4:** **Medial heel**

***Statistical values***	***Contact area*** **(** ***CA*** **)/** ***Pressure*** **(** ***P*** **)**		**Contact area (CA)/Force (F)**	
	**Left**	**Right**	**Left**	**Right**
Media	67.85	68.7	69.25	67.695
Standard deviation (SD)	14.66566	17.83875	14.35957	23.06096
Min	27	39	29	6.9
Max	96	100	96	100
No. values	20	20	20	20
***P*** **(Student’s** ***t*** **) for contact area (CA)**	0.4005245	0.41909	0.339318	0.63092

**Table 5:** **Midfoot**

***Statistical values***	***Contact area*** **(** ***CA*** **)/** ***Pressure*** **(** ***P*** **)**		**Contact area (CA)/Force (F)**	
	**Left**	**Right**	**Left**	**Right**
Media	76.5	66.4	77.45	81.425
Standard deviation (SD)	11.21324	18.18733	14.31405	50.36584
Min	58	30	30	34
Max	96	96	98	281
No. values	20	20	20	20
***P*** **(Student’s** ***t*** **) for contact area (CA)**	0.073529	0.974372	0.241004	0.404075

**Table 6:** **Toes II to V**

***Statistical values***	***Contact area*** **(** ***CA*** **)/** ***Pressure*** **(** ***P*** **)**		**Contact area (CA)/Force (F)**	
	**Left**	**Right**	**Left**	**Right**
Media	50.5	57.3	54	62.34
Standard deviation (SD)	27.3313	28.45329	28.43275	32.56243
Min	18	4	19	5
Max	99	100	99	133.8
No. values	20	20	20	20
***P*** **(Student’s** ***t*** **) for contact area (CA)**	0.437635	0.425615	0.307645	0.269141

**Table 7:** **Contact area**

	***Average (cm*** ^***2***^ ***)***	
	**Left**	**Right**
Lateral heel	67.35	65.52
Medial heel	68.55	68.19
Midfoot	76.97	73.91
Toes II to V	33	59.82

The impulse parameter results and the related statistical analyses are presented in Tables 8, 9, 10, 11, reporting pressure and force. The average values for impulse are presented in Table 12.

**Table 8:** **Lateral heel**

***Statistical values***	***Impulse*** **(** ***I*** **)/** ***Pressure*** **(** ***P*** **)**		**Impulse (I)/Force (F)**	
	**Left**	**Right**	**Left**	**Right**
Media	6.747368	6.455	111.0421	109.445
Standard deviation (SD)	6.546871	5.067385	113.1596	92.12411
Min	0.9	0.9	5.7	8.6
Max	30.4	19.8	512.6	337.4
No. values	19	20	19	20
***P*** **(Student’s** ***t*** **) for contact area (CA)**	0.028479	0.063035	0.037838	0.104032

**Table 9:** **Medial heel**

***Statistical values***	***Impulse*** **(** ***I*** **)/** ***Pressure*** **(** ***P*** **)**		**Impulse (I)/Force (F)**	
	**Left**	**Right**	**Left**	**Right**
Media	6.742105	6.589474	127.1684	138.0316
Standard deviation (SD)	6.051475	5.147696	111.7632	123.133
Min	0.6	1.3	9.3	22
Max	29.2	18.1	535.7	400.5
No. values	19	19	19	19
***P*** **(Student’s** ***t*** **) for contact area (CA)**	0.187849	0.290577	0.150613	0.30166

**Table 10:** **Midfoot**

***Statistical values***	***Impulse*** **(** ***I*** **)/** ***Pressure*** **(** ***P*** **)**		**Impulse (I)/Force (F)**	
	**Left**	**Right**	**Left**	**Right**
Media	3.063158	3.55	138.7895	107.19
Standard deviation (SD)	3.720246	7.954972	156.8798	107.19
Min	0.8	0.4	18.7	13.2
Max	16.9	37	682.5	791.4
No. values	19	20	19	20
***P*** **(Student’s** ***t*** **) for contact area (CA)**	0.38124	0.304365	0.324948	0.449568

**Table 11:** **Toes II to V**

***Statistical values***	***Impulse*** **(** ***I*** **)/** ***Pressure*** **(** ***P*** **)**		**Impulse (I)/Force (F)**	
	**Left**	**Right**	**Left**	**Right**
Media	1.188889	2.111111	12.11667	29.15789
Standard deviation (SD)	1.432353	2.594766	20.30619	37.6766
Min	0.1	0.1	0.4	0.4
Max	5.2	10.7	71.4	161
No. values	18	18	18	19
***P*** **(Student’s** ***t*** **) for contact area (CA)**	0.183159	0.326649	0.938496	0.343283

**Table 12:** **Average values for impulse**

	***Average Ns/cm***	
	**Left**	**Right**
Lateral heel	58.89	57.94
Medial heel	66.95	72.30
Midfoot	70.92	55.37
Toes II to V	13.29	15.63

Loading is a kinematic parameter that allows us to approach how the load of the plantar region is made and how proprioceptive stimulation occurs. We recorded the same regions of the plantar side and the results are presented in Tables 13, 14, 15, 16. The average values for loading are presented in Table 17.

**Table 13:** **Lateral heel**

***Statistical values***	***Loading*** **(** ***L*** **)/** ***Pressure*** **(** ***P*** **)**		**Loading (L)/Force (F)**	
	**Left**	**Right**	**Left**	**Right**
Media	0.4785	0.4105	7.1745	7.7475
Standard deviation (SD)	1.304653	0.701363	22.55043	14.25034
Min	0.01	0.02	0.05	0.14
Max	5.91	2.74	101.95	58.25
No. values	20	20	20	20
***P*** **(Student’s** ***t*** **) for contact area (CA)**	0.795654	0.750699	0.79095	0.857492

**Table 14:** **Medial heel**

***Statistical values***	***Impulse*** **(** ***I*** **)/** ***Pressure*** **(** ***P*** **)**		**Impulse (I)/Force (F)**	
	**Left**	**Right**	**Left**	**Right**
Media	0.273158	0.109474	4.314211	2.133158
Standard deviation (SD)	0.489933	0.088599	9.637259	1.759381
Min	0.01	0.01	0.07	0.09
Max	2.05	0.3	43.1	5.51
No. values	19	19	19	19
***P*** **(Student’s** ***t*** **) for contact area (CA)**	0.643494	0.051056	0.525328	0.065216

**Table 15:** **Midfoot**

***Statistical values***	***Impulse*** **(** ***I*** **)/** ***Pressure*** **(** ***P*** **)**		**Impulse (I)/Force (F)**	
	**Left**	**Right**	**Left**	**Right**
Media	0.027778	0.027059	1.0105	0.778
Standard deviation (SD)	0.023151	0.017594	0.927682	0.637137
Min	0.01	0.01	0.1	0.1
Max	0.1	0.06	3.57	1.9
No. values	18	17	20	20
***P*** **(Student’s** ***t*** **) for contact area (CA)**	0.018593	0.081247	0.039268	0.040208

**Table 16:** **Toes II to V**

***Statistical values***	***Impulse*** **(** ***I*** **)/** ***Pressure*** **(** ***P*** **)**		**Impulse (I)/Force (F)**	
	**Left**	**Right**	**Left**	**Right**
Media	0.0225	0.02	0.161176	0.208889
Standard deviation (SD)	0.015275	0.013628	0.137244	0.140541
Min	0.01	0.01	0.01	0.03
Max	0.05	0.05	0.43	0.5
No. values	16	15	**18**	18
***P*** **(Student’s** ***t*** **) for contact area (CA)**	0.056463	0.386342	0.747686	0.384808

**Table 17:** **Average values for loading**

	***Average N/cms***	
	**Left**	**Right**
Lateral heel	3.86	2.53
Medial heel	6.13	2.80
Midfoot	2.46	1.07
Toes II to V	0.11	0.17

### Foot balance

Even if patients have not got a clinical aspect of gait disorder, we can assess this by evaluating the foot balance. We observe that it is in all gait phases because the foot is oriented on exorotation and supination average values −59° and −10° (negative values meaning exorotation and supination).

### Load of metatarsal region

The patients with clinical gait disorders have foot stability in heel contact and the stance phase, but an increase of instability during the swing phase and also a great asymmetry right to left.

### Distribution of pressure centre (CP)

In most patients we observed a great asymmetry of pressure centre (CP), and it was from the anterior to the posterior side, which is high for the medial heel for one foot, and metatarsian I to III and toe I, for the other foot. In patients without clinical manifestation of gait disorders, we observed an increase of CP distribution on metatarsian II to III and less on the heel.

### Subtalar angle

This angle is important for ankle and foot stability and it has a minimum average value of −1.18° for the right foot and −4.27° for the left foot, and for a maximum average value of 6.27° for the right foot and 5.03° for the left foot. These values show us that the evolution of values is from negative to positive during the gait phase.

## Discussion

This study shows that the major lesion is to the pyramidal system and the average value for functionality index (score EDSS) is 3.27 ± 0.15, where 3 means easy paraparesis or hemiparesis. A total of 52% of the patients scored 1 to 3 points, indicating minimal handicap, without visible clinical gait disorders. EDSS scores of more than 5 for 23 patients (48% of patients) meaning assistance was needed in daily activities for 16 subjects (assistance and stance devices) including dependence on a wheelchair, and 7 subjects had no ambulation.

Evaluation of gait and motor performance is a function of the management of MS.

Even if in their daily assessments many physicians and physical therapists use a functional scale assessment like a walking test of 6 m or 10 m, it does not help us achieve a complex assessment of gait and lower limb behaviour during gait , and provides no information about the impact of gait on daily activities and quality of life [18] .

The recent meeting of the Consortium of Multiple Sclerosis Centers (CMSC) included a discussion regarding a proposed protocol for the complex assessment of gait in MS [19], showing that methods for this kind of assessment could give important information about gait features in MS that could be useful for the design of a rehabilitation process, and for testing their contribution, but, in any case, they are hard to apply in clinical practice. A new approach is to use the movement system analysis, like the SIMI Motion system (Simi, Unterschleißheim, Germany) associated with a force plate [18], for qualitative evaluation. Quantitative evaluation can be detected in the early stage of gait disorders, even if there is a lack of clinical disorders using complex assessment that combine clinical assessment and biomechanical assessment.

One of the aspects is asymmetry of the gait, even in normal gait, and much more so in MS [20], which can be explained by a functional discrepancy of the load on lower limbs that could influence foot control and the swing phase. One of the legs is responsible for control and weight support and the other leg has a role in the swing phase [20,21]. In our study, we concluded that local asymmetry is a significant statistic for gait and for comparison between the right and left foot, which is in accordance with the Sadeghi study [20].

In our research, we made a biomechanical evaluation using the RSscan force plate from the aspect of the importance of kinetic analysis of gait based on force, pressure and other parameters, which present changes during gait phases. Ground reaction force (GRF) comes to ankle, foot, knee and hip and its evolution respects the following equations:

$$ \mathrm{P}\kern0.5em =\kern0.5em \mathrm{F}/\mathrm{S}\mathrm{c},\ \mathrm{P}\kern0.5em =\kern0.5em \mathrm{pressure},\ \mathrm{F} = \mathrm{force}\ \left(\mathrm{b}\mathrm{y}\ \mathrm{body}\ \mathrm{weight}\right),\ \mathrm{S}\mathrm{c} = \mathrm{c}\mathrm{ontact}\ \mathrm{surface} $$

Based on this aspect, all information can be useful for designing a rehabilitation programme, regarding foot and ankle load, motor control and the coordination of movement. By this evaluation we see that it is possible to record the distribution of pressure at the plantar region in relation to weight and force, which helps us obtain a fast analysis of foot movement from the beginning of the gait cycle. This explains why many MS patients have gait disorders because of demylinisation, which involves neurologic disorders, and the impact under the proprioceptive system. Our observation is in accordance with Fjeldstad [22], who says that specific evaluation testing of foot balance allows an estimate of the proprioceptive system, which is the most affected in MS.

From this point of view, one parameter is CA, which has differences between the right and left foot at the lateral heel, and differences of maximum and minimum values, which means a tendency to increase the CA-like compensation mechanism for balance and stability. The same tendency applies to the medial heel produced by lack of foot motor control in the heel contact gait phase and because of a compensation mechanism for increasing balance.

On the other side, we observe that there is a functional deficit of the ankle joint, which involves changes of the physiological direction of force from shank to foot, meaning a decomposition on the vertical and horizontal planes. As Houglum [23] observes, we demonstrated an increase of dorsiflexor action to prevent the foot fall, that involves tibialis anterior (TA) contraction, which is specific in MS, and the increase of ankle instability produced by gastrocnemius muscles.

Impulse is another parameter that has high values in MS patients because of less motor control. This aspect is also present [24], which suggests the decrease of plantar flexion and of force propulsion in the swing phase, demonstrated by dynamic electromyography (EMG) in this study.

Loading is a kinematic parameter related to the muscle force and impulse that helps us to understand proprioceptive stimulation and motor control. Our analysis shows that there exists a large zone for minimum and maximum values for loading at the heel region (0.01/101.95 to 0.01/43.1) in the case of MS patients when compared with healthy people who have a maximum value of 15.5 and minimum value of 0.2.

This means that in MS patients, the loading at the plantar region is high, which might be due to balance disorders. At midfoot, the values are low for both feet but without asymmetry. Also we observed that in this region right to left asymmetry is not significant, meaning that it is not a distribution of force or pressure in this region, because the high distribution is on the heel, keeping the mass centred inside the support area.

Regarding toes II to V, we note the high values of loading because of balance disorders during gait, which can be explained by an adapting mechanism like anteroposterior recovery.

There are two aspects of heel rotation and foot evolution: heel contact and exorotation (supination): the foot will go on pronation and again on supination for one leg, while the other leg goes from pronation to a neutral position. Both can be explained by an adaptative mechanism of motor control to restore the balance.

Foot balance is a feature of the foot, especially for the swing phase because it influences the heel contact in the next gait phase and is related to heel exorotation. Foot balance disorders are present in all MS patients even if the clinical disorders do not exist, and are present in all gait phases, and are associated with lack of a neutral foot position.

These disorders are different left to right, because supination is for one leg while pronation is for the opposite leg.

The load of the metatarsian region helps us to estimate the motor control of the foot and the weight distribution from left to right during gait. This aspect is important for ankle and foot stability and depends on how the heel rotation and foot balance are.

We observed a left to right instability to be more evident in the swing phase and its influence on the foot impulse for the next step and for postural control, when considering postural strategies. Our observation is based on the high impulse values on the left lateral heel, because, during the swing phase, foot motor control is not possible, which influences the impulse on the next initial contact, causing an abnormal contact of the heel and transfer to midstance. This impulse will be higher than normal and it will be necessary to develop an ankle posture strategy from the other leg, to maintain balance and stability.

From the analysis of the data regarding the pressure centre position, we can see that the high pressure is on metatarsian II to III and more or less at the heel. This implies development of an ankle strategy is necessary to restore balance, stability and motor control, which cannot be assessed other than by clinical evaluation.

In analysing the foot position in relation to the longitudinal axis of the body, we observed that foot abduction is related to an increase in pronation and a reduction in the contact area for the midfoot.

Regarding the evaluation of the foot for MS patients who have no clinical signs for gait disorders, Benedetti [24] made gait assessments using parameters such as: time of step, speed, length of step, and concluded that all changes are based on motor changes that develop before the clinical signs start. This is in accordance with our conclusions regarding the importance of biomechanical assessment of the foot for estimating gait in MS. Much more so if we think of the pathogenic mechanism, we can say that all these changes are the result of a delay in the nervous system transmissions that involve foot motor control disorders.

## Conclusions

Biomechanical assessment can help the clinician predict the functional evolution of MS patients without clinical gait disorders and allows the development of strategies for rehabilitation that prevent the incorrect positioning of the ankle and foot, produced by the lack of motor control.

Clinicians have the possibility of monitoring the ankle and foot function during static and dynamic balance, and gait and enabling proprioceptive training to enhance the afferents (inputs) to the central nervous system and develop some way to increase motor control of the foot.

Even if patients have a clinically normal gait (MS subjects without visible clinical gait disorders), we can demonstrate that there exists an abnormal asymmetry of gait, load and distribution of pressure centre, making it possible to predict the evolution of MS and its impact on functional deficiency.

Using a complex assessment, a clinician can identify the appropriate time for rehabilitation as a prophylactic means for the prevention of tissue destruction, and for training the muscle adaptative mechanism, based on muscle composition and muscle plasticity of skeletal muscle.

## Abbreviations

CA: contact area
CMSC: Consortium of Multiple Sclerosis Centers
CP: distribution of pressure centre
EDSS/ Kurtzke: Expanded Disability Status Scale
EMG: electromyography
F: force
GRF: ground reaction force
HL: lateral heel
HM: medial heel
I: impulse
IDH: Impairment, Disabilities, Handicap Scale
L: loading
LR: load rate
MF: midfoot
MRI: magnetic resonance imaging
MMSE: mini-mental state examination
MS: multiple sclerosis
P: pressure
PPMS: primary progressive multiple sclerosis
Sc: contact surface
TA: tibialis anterior
